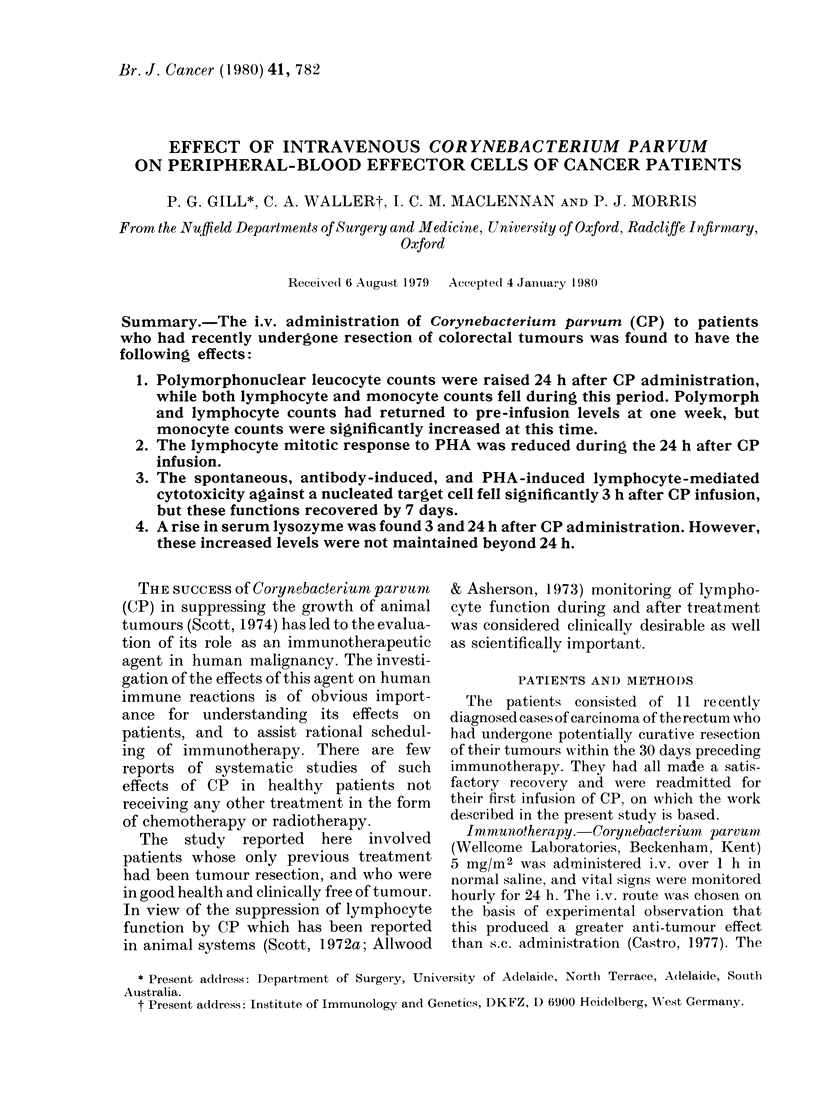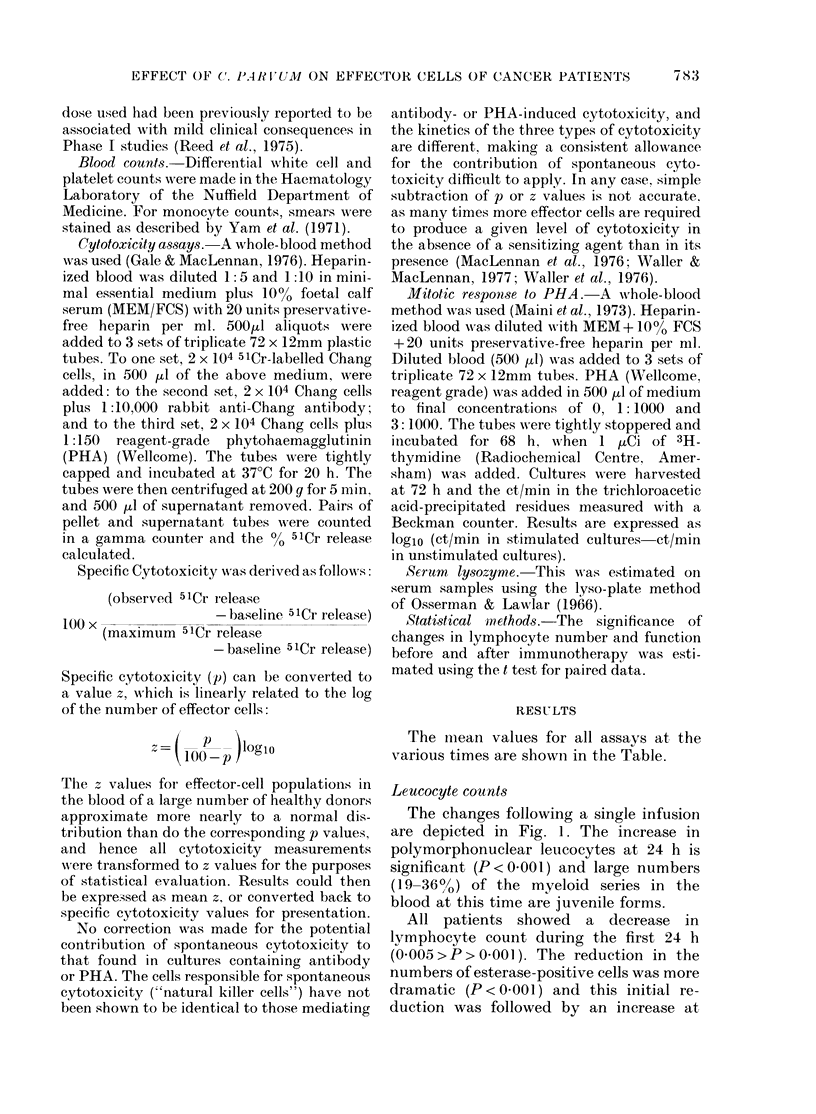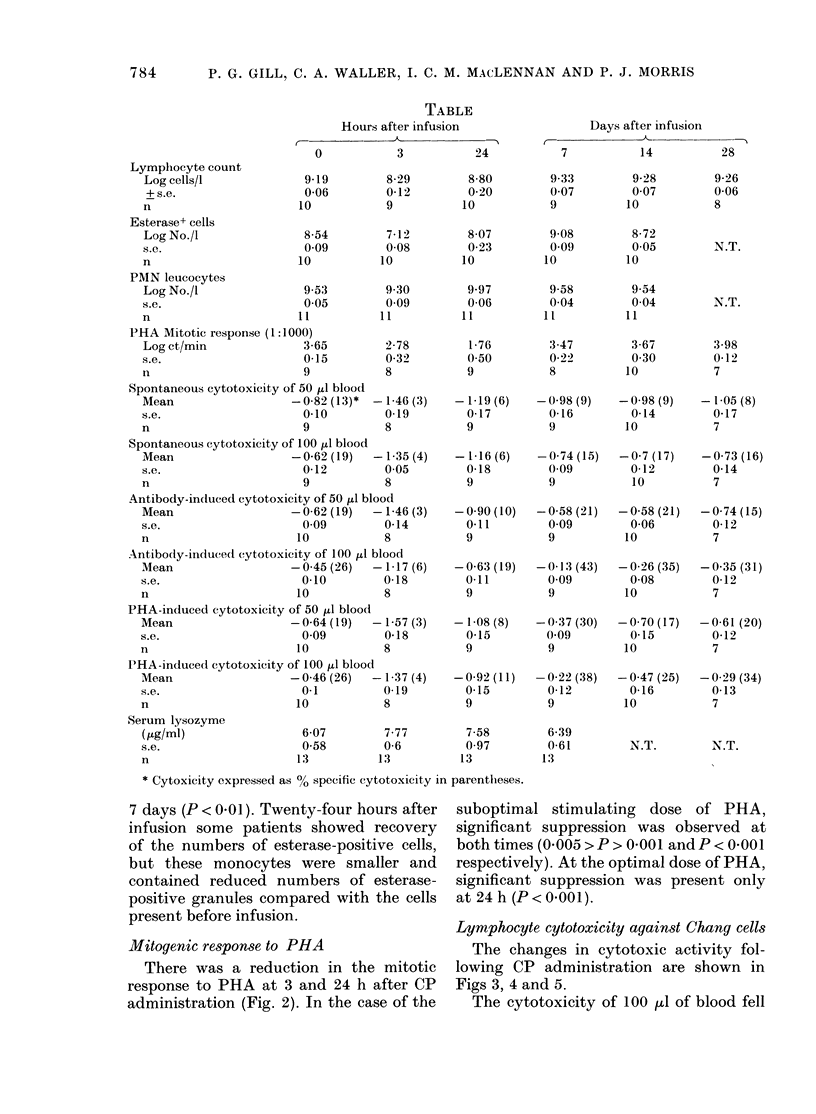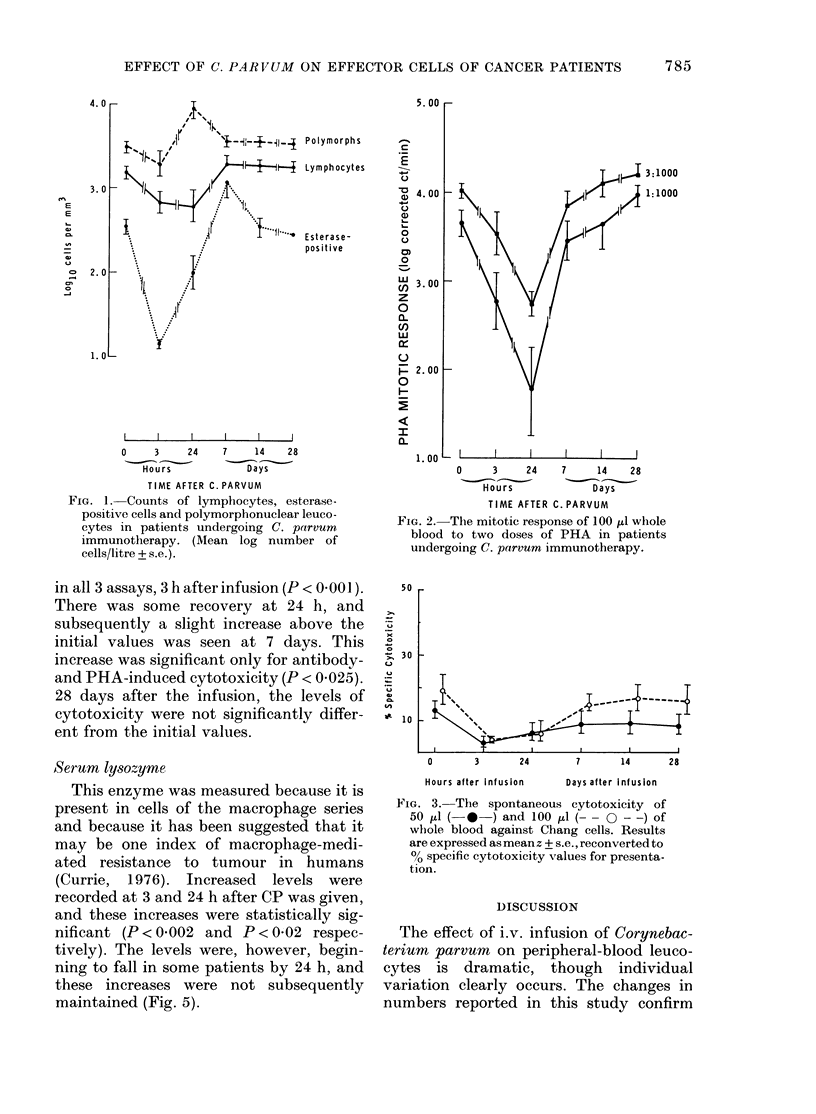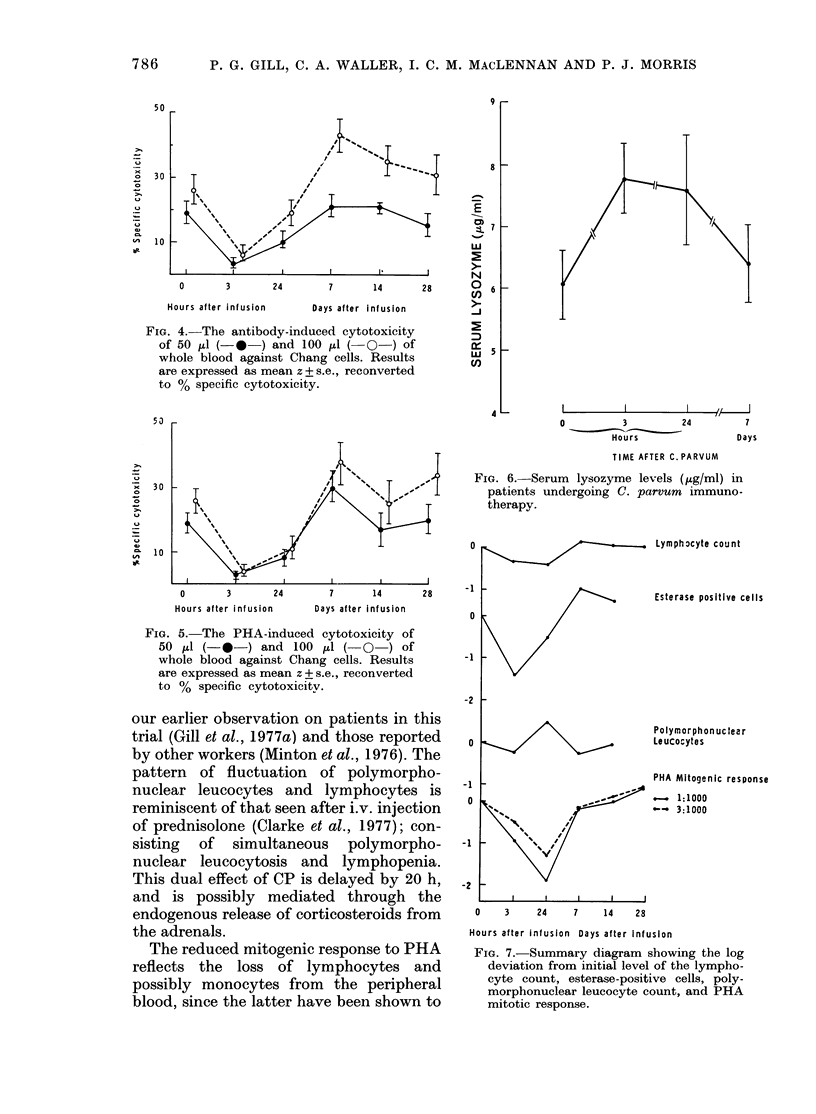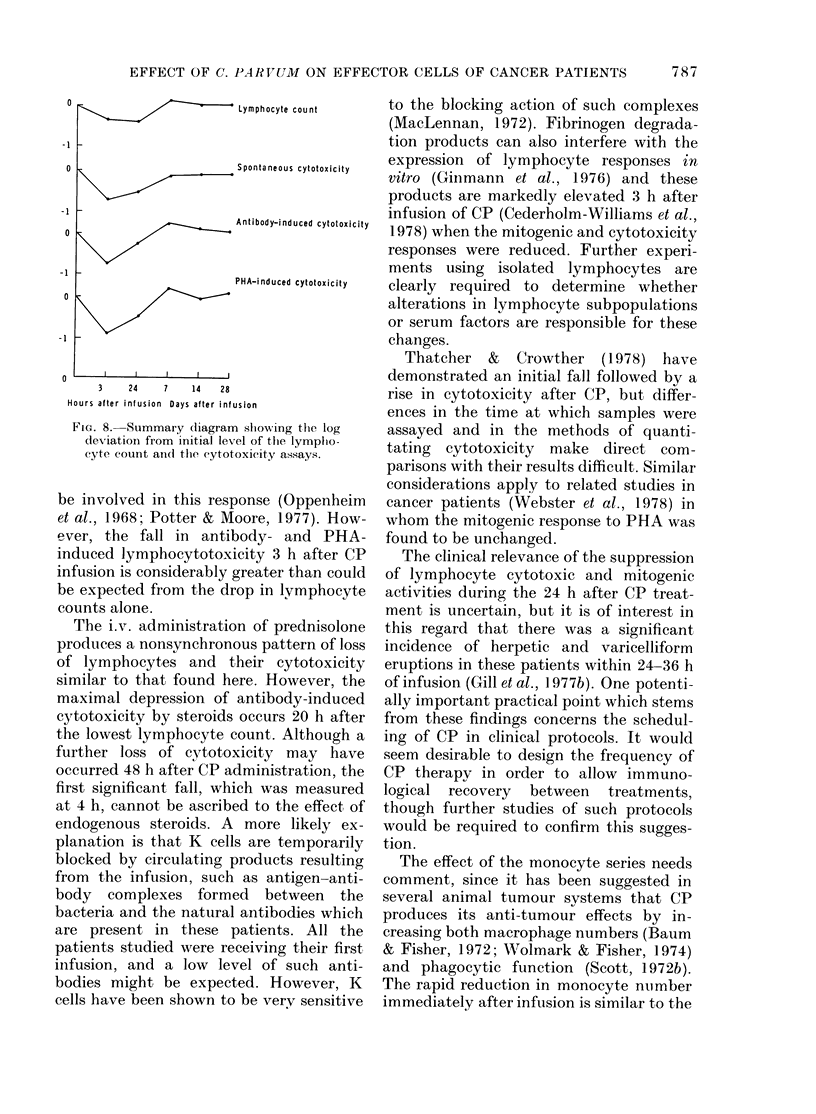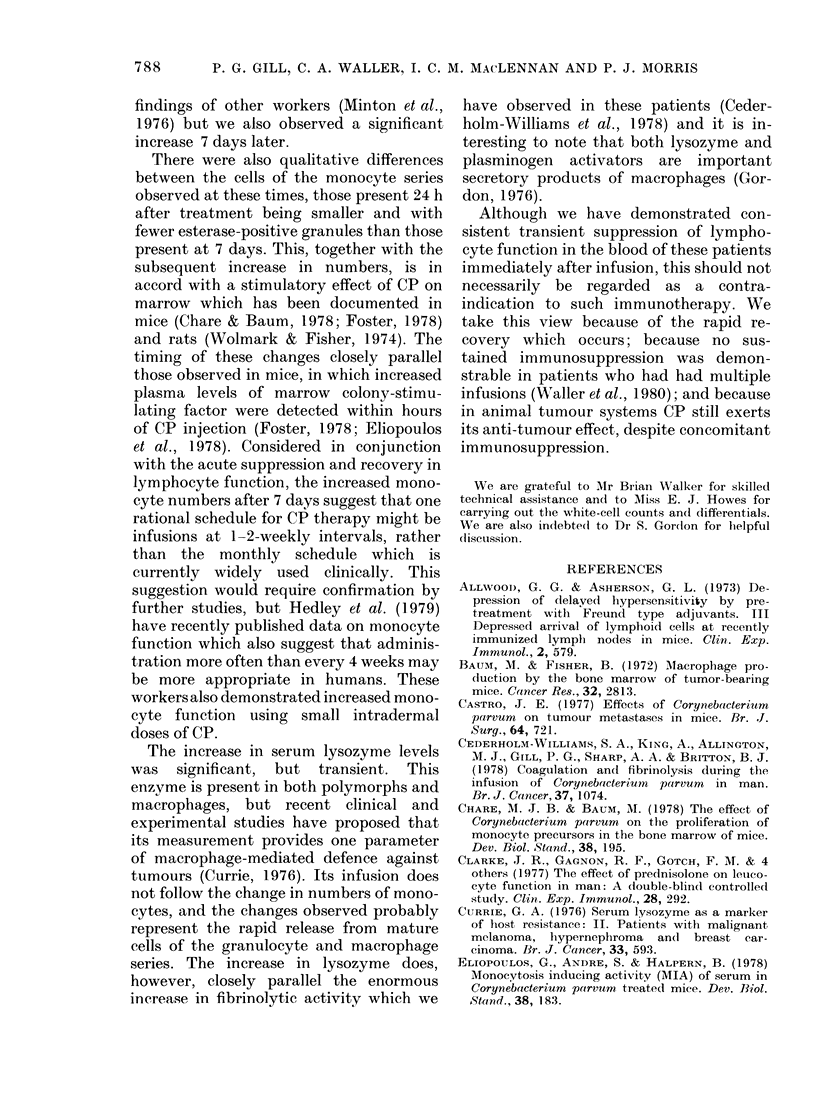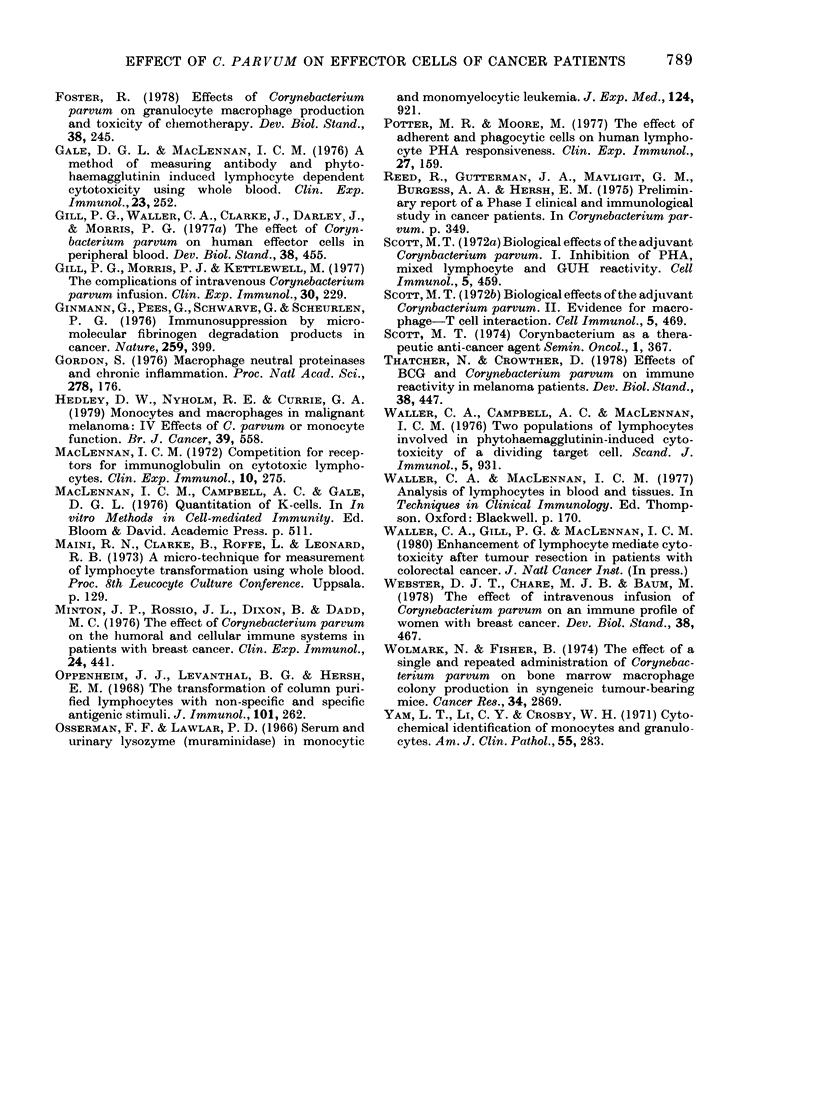# Effect of intravenous corynebacterium parvum on peripheral-blood effector cells of cancer patients.

**DOI:** 10.1038/bjc.1980.142

**Published:** 1980-05

**Authors:** P. G. Gill, C. A. Waller, I. C. MacClennan, P. J. Morris

## Abstract

The i.v. administration of Corynebacterium parvum (CP) to patients who had recently undergone resection of colorectal tumours was found to have the following effects: 1. Polymorphonuclear leucocyte counts were raised 24 h after CP administration, while both lymphocyte and monocyte counts fell during this period. Polymorph and lymphocyte counts had returned to pre-infusion levels at one week, but monocyte counts were significantly increased at this time. 2. The lymphocyte mitotic response to PHA was reduced during the 24 h after CP infusion. 3 The spontaneous, antibody-induced, and PHA-induced lymphocyte-mediated cytotoxicity against a nucleated target cell fell significantly 3 h after CP infusion, but these functions recovered by 7 days. 4. A rise in serum lysozyme was found 3 and 24 h after CP administration. However, these increased levels were not maintained beyond 24 h.


*Br. J. Cancer* (1980) **41**, 782

### EFFECT OF INTRAVENOUS *CORYNEBACTERIUM PARVUM* ON PERIPHERAL-BLOOD EFFECTOR CELLS OF CANCER PATIENTS

P. G. GILL*, C. A. WALLER†, I. C. M. MACLENNAN AND P. J. MORRIS

*From the Nuffield Departments of Surgery and Medicine, University of Oxford, Radcliffe Infirmary, Oxford*

Received 6 August 1979 Accepted 4 January 1980

**Summary.—The i.v. administration of *Corynebacterium parvum* (CP) to patients who had recently undergone resection of colorectal tumours was found to have the following effects:**

1. **Polymorphonuclear leucocyte counts were raised 24 h after CP administration, while both lymphocyte and monocyte counts fell during this period. Polymorph and lymphocyte counts had returned to pre-infusion levels at one week, but monocyte counts were significantly increased at this time.**
2. **The lymphocyte mitotic response to PHA was reduced during the 24 h after CP infusion.**
3. **The spontaneous, antibody-induced, and PHA-induced lymphocyte-mediated cytotoxicity against a nucleated target cell fell significantly 3 h after CP infusion, but these functions recovered by 7 days.**
4. **A rise in serum lysozyme was found 3 and 24 h after CP administration. However, these increased levels were not maintained beyond 24 h.**

THE SUCCESS of *Corynebacterium parvum* (CP) in suppressing the growth of animal tumours (Scott, 1974) has led to the evaluation of its role as an immunotherapeutic agent in human malignancy. The investigation of the effects of this agent on human immune reactions is of obvious importance for understanding its effects on patients, and to assist rational scheduling of immunotherapy. There are few reports of systematic studies of such effects of CP in healthy patients not receiving any other treatment in the form of chemotherapy or radiotherapy.

The study reported here involved patients whose only previous treatment had been tumour resection, and who were in good health and clinically free of tumour. In view of the suppression of lymphocyte function by CP which has been reported in animal systems (Scott, 1972*a*; Allwood & Asherson, 1973) monitoring of lymphocyte function during and after treatment was considered clinically desirable as well as scientifically important.

### PATIENTS AND METHODS

The patients consisted of 11 recently diagnosed cases of carcinoma of the rectum who had undergone potentially curative resection of their tumours within the 30 days preceding immunotherapy. They had all made a satisfactory recovery and were readmitted for their first infusion of CP, on which the work described in the present study is based.

*Immunotherapy.—Corynebacterium parvum* (Wellcome Laboratories, Beckenham, Kent) 5 mg/m$^2$ was administered i.v. over 1 h in normal saline, and vital signs were monitored hourly for 24 h. The i.v. route was chosen on the basis of experimental observation that this produced a greater anti-tumour effect than s.c. administration (Castro, 1977). The

* Present address: Department of Surgery, University of Adelaide, North Terrace, Adelaide, South Australia.

† Present address: Institute of Immunology and Genetics, DKFZ, D 6900 Heidelberg, West Germany.

dose used had been previously reported to be associated with mild clinical consequences in Phase I studies (Reed *et al.*, 1975).

*Blood counts.*—Differential white cell and platelet counts were made in the Haematology Laboratory of the Nuffield Department of Medicine. For monocyte counts, smears were stained as described by Yam *et al.* (1971).

*Cytotoxicity assays.*—A whole-blood method was used (Gale & MacLennan, 1976). Heparinized blood was diluted 1:5 and 1:10 in minimal essential medium plus 10% foetal calf serum (MEM/FCS) with 20 units preservative-free heparin per ml. 500$\mu$l aliquots were added to 3 sets of triplicate 72 × 12mm plastic tubes. To one set, $2 \times 10^4$ $^{51}Cr$-labelled Chang cells, in 500 $\mu$l of the above medium, were added: to the second set, $2 \times 10^4$ Chang cells plus 1:10,000 rabbit anti-Chang antibody; and to the third set, $2 \times 10^4$ Chang cells plus 1:150 reagent-grade phytohaemagglutinin (PHA) (Wellcome). The tubes were tightly capped and incubated at 37°C for 20 h. The tubes were then centrifuged at 200 *g* for 5 min, and 500 $\mu$l of supernatant removed. Pairs of pellet and supernatant tubes were counted in a gamma counter and the % $^{51}Cr$ release calculated.

Specific Cytotoxicity was derived as follows:

$$100 \times \frac{(\text{observed } ^{51}\text{Cr release} - \text{baseline } ^{51}\text{Cr release})}{(\text{maximum } ^{51}\text{Cr release} - \text{baseline } ^{51}\text{Cr release})}$$

Specific cytotoxicity ($p$) can be converted to a value $z$, which is linearly related to the log of the number of effector cells:

$$z = \left(\frac{p}{100-p}\right)\log_{10}$$

The $z$ values for effector-cell populations in the blood of a large number of healthy donors approximate more nearly to a normal distribution than do the corresponding $p$ values, and hence all cytotoxicity measurements were transformed to $z$ values for the purposes of statistical evaluation. Results could then be expressed as mean $z$, or converted back to specific cytotoxicity values for presentation.

No correction was made for the potential contribution of spontaneous cytotoxicity to that found in cultures containing antibody or PHA. The cells responsible for spontaneous cytotoxicity ("natural killer cells") have not been shown to be identical to those mediating antibody- or PHA-induced cytotoxicity, and the kinetics of the three types of cytotoxicity are different, making a consistent allowance for the contribution of spontaneous cytotoxicity difficult to apply. In any case, simple subtraction of $p$ or $z$ values is not accurate, as many times more effector cells are required to produce a given level of cytotoxicity in the absence of a sensitizing agent than in its presence (MacLennan *et al.*, 1976; Waller & MacLennan, 1977; Waller *et al.*, 1976).

*Mitotic response to PHA.*—A whole-blood method was used (Maini *et al.*, 1973). Heparinized blood was diluted with MEM + 10% FCS + 20 units preservative-free heparin per ml. Diluted blood (500 $\mu$l) was added to 3 sets of triplicate 72 × 12mm tubes. PHA (Wellcome, reagent grade) was added in 500 $\mu$l of medium to final concentrations of 0, 1:1000 and 3:1000. The tubes were tightly stoppered and incubated for 68 h, when 1 $\mu$Ci of $^3H$-thymidine (Radiochemical Centre, Amersham) was added. Cultures were harvested at 72 h and the ct/min in the trichloroacetic acid-precipitated residues measured with a Beckman counter. Results are expressed as $\log_{10}$ (ct/min in stimulated cultures—ct/min in unstimulated cultures).

*Serum lysozyme.*—This was estimated on serum samples using the lyso-plate method of Osserman & Lawlar (1966).

*Statistical methods.*—The significance of changes in lymphocyte number and function before and after immunotherapy was estimated using the $t$ test for paired data.

### RESULTS

The mean values for all assays at the various times are shown in the Table.

### *Leucocyte counts*

The changes following a single infusion are depicted in Fig. 1. The increase in polymorphonuclear leucocytes at 24 h is significant ($P < 0{\cdot}001$) and large numbers (19–36%) of the myeloid series in the blood at this time are juvenile forms.

All patients showed a decrease in lymphocyte count during the first 24 h ($0{\cdot}005 > P > 0{\cdot}001$). The reduction in the numbers of esterase-positive cells was more dramatic ($P < 0{\cdot}001$) and this initial reduction was followed by an increase at

TABLE

| | Hours after infusion | | | Days after infusion | | |
|---|---|---|---|---|---|---|
| | 0 | 3 | 24 | 7 | 14 | 28 |
| Lymphocyte count | | | | | | |
| Log cells/l | 9·19 | 8·29 | 8·80 | 9·33 | 9·28 | 9·26 |
| ± s.e. | 0·06 | 0·12 | 0·20 | 0·07 | 0·07 | 0·06 |
| n | 10 | 9 | 10 | 9 | 10 | 8 |
| Esterase+ cells | | | | | | |
| Log No./l | 8·54 | 7·12 | 8·07 | 9·08 | 8·72 | |
| s.e. | 0·09 | 0·08 | 0·23 | 0·09 | 0·05 | N.T. |
| n | 10 | 10 | 10 | 10 | 10 | |
| PMN leucocytes | | | | | | |
| Log No./l | 9·53 | 9·30 | 9·97 | 9·58 | 9·54 | |
| s.e. | 0·05 | 0·09 | 0·06 | 0·04 | 0·04 | N.T. |
| n | 11 | 11 | 11 | 11 | 11 | |
| PHA Mitotic response (1:1000) | | | | | | |
| Log ct/min | 3·65 | 2·78 | 1·76 | 3·47 | 3·67 | 3·98 |
| s.e. | 0·15 | 0·32 | 0·50 | 0·22 | 0·30 | 0·12 |
| n | 9 | 8 | 9 | 8 | 10 | 7 |
| Spontaneous cytotoxicity of 50 μl blood | | | | | | |
| Mean | −0·82 (13)* | −1·46 (3) | −1·19 (6) | −0·98 (9) | −0·98 (9) | −1·05 (8) |
| s.e. | 0·10 | 0·19 | 0·17 | 0·16 | 0·14 | 0·17 |
| n | 9 | 8 | 9 | 9 | 10 | 7 |
| Spontaneous cytotoxicity of 100 μl blood | | | | | | |
| Mean | −0·62 (19) | −1·35 (4) | −1·16 (6) | −0·74 (15) | −0·7 (17) | −0·73 (16) |
| s.e. | 0·12 | 0·05 | 0·18 | 0·09 | 0·12 | 0·14 |
| n | 9 | 8 | 9 | 9 | 10 | 7 |
| Antibody-induced cytotoxicity of 50 μl blood | | | | | | |
| Mean | −0·62 (19) | −1·46 (3) | −0·90 (10) | −0·58 (21) | −0·58 (21) | −0·74 (15) |
| s.e. | 0·09 | 0·14 | 0·11 | 0·09 | 0·06 | 0·12 |
| n | 10 | 8 | 9 | 9 | 10 | 7 |
| Antibody-induced cytotoxicity of 100 μl blood | | | | | | |
| Mean | −0·45 (26) | −1·17 (6) | −0·63 (19) | −0·13 (43) | −0·26 (35) | −0·35 (31) |
| s.e. | 0·10 | 0·18 | 0·11 | 0·09 | 0·08 | 0·12 |
| n | 10 | 8 | 9 | 9 | 10 | 7 |
| PHA-induced cytotoxicity of 50 μl blood | | | | | | |
| Mean | −0·64 (19) | −1·57 (3) | −1·08 (8) | −0·37 (30) | −0·70 (17) | −0·61 (20) |
| s.e. | 0·09 | 0·18 | 0·15 | 0·09 | 0·15 | 0·12 |
| n | 10 | 8 | 9 | 9 | 10 | 7 |
| PHA-induced cytotoxicity of 100 μl blood | | | | | | |
| Mean | −0·46 (26) | −1·37 (4) | −0·92 (11) | −0·22 (38) | −0·47 (25) | −0·29 (34) |
| s.e. | 0·1 | 0·19 | 0·15 | 0·12 | 0·16 | 0·13 |
| n | 10 | 8 | 9 | 9 | 10 | 7 |
| Serum lysozyme | | | | | | |
| (μg/ml) | 6·07 | 7·77 | 7·58 | 6·39 | | |
| s.e. | 0·58 | 0·6 | 0·97 | 0·61 | N.T. | N.T. |
| n | 13 | 13 | 13 | 13 | | |

* Cytoxicity expressed as % specific cytotoxicity in parentheses.

7 days ($P < 0{\cdot}01$). Twenty-four hours after infusion some patients showed recovery of the numbers of esterase-positive cells, but these monocytes were smaller and contained reduced numbers of esterase-positive granules compared with the cells present before infusion.

*Mitogenic response to PHA*

There was a reduction in the mitotic response to PHA at 3 and 24 h after CP administration (Fig. 2). In the case of the suboptimal stimulating dose of PHA, significant suppression was observed at both times ($0{\cdot}005 > P > 0{\cdot}001$ and $P < 0{\cdot}001$ respectively). At the optimal dose of PHA, significant suppression was present only at 24 h ($P < 0{\cdot}001$).

*Lymphocyte cytotoxicity against Chang cells*

The changes in cytotoxic activity following CP administration are shown in Figs 3, 4 and 5.

The cytotoxicity of 100 μl of blood fell

FIG. 1.—Counts of lymphocytes, esterase-positive cells and polymorphonuclear leucocytes in patients undergoing *C. parvum* immunotherapy. (Mean log number of cells/litre ± s.e.).

FIG. 2.—The mitotic response of 100 μl whole blood to two doses of PHA in patients undergoing *C. parvum* immunotherapy.

in all 3 assays, 3 h after infusion ($P < 0{\cdot}001$). There was some recovery at 24 h, and subsequently a slight increase above the initial values was seen at 7 days. This increase was significant only for antibody- and PHA-induced cytotoxicity ($P < 0{\cdot}025$). 28 days after the infusion, the levels of cytotoxicity were not significantly different from the initial values.

*Serum lysozyme*

This enzyme was measured because it is present in cells of the macrophage series and because it has been suggested that it may be one index of macrophage-mediated resistance to tumour in humans (Currie, 1976). Increased levels were recorded at 3 and 24 h after CP was given, and these increases were statistically significant ($P < 0{\cdot}002$ and $P < 0{\cdot}02$ respectively). The levels were, however, beginning to fall in some patients by 24 h, and these increases were not subsequently maintained (Fig. 5).

FIG. 3.—The spontaneous cytotoxicity of 50 μl (—●—) and 100 μl (– – ○ – –) of whole blood against Chang cells. Results are expressed as mean $z$ ± s.e., reconverted to % specific cytotoxicity values for presentation.

DISCUSSION

The effect of i.v. infusion of *Corynebacterium parvum* on peripheral-blood leucocytes is dramatic, though individual variation clearly occurs. The changes in numbers reported in this study confirm

FIG. 4.—The antibody-induced cytotoxicity of 50 μl (—●—) and 100 μl (—○—) of whole blood against Chang cells. Results are expressed as mean $z \pm$ s.e., reconverted to % specific cytotoxicity.

FIG. 5.—The PHA-induced cytotoxicity of 50 μl (—●—) and 100 μl (—○—) of whole blood against Chang cells. Results are expressed as mean $z \pm$ s.e., reconverted to % specific cytotoxicity.

FIG. 6.—Serum lysozyme levels (μg/ml) in patients undergoing *C. parvum* immunotherapy.

FIG. 7.—Summary diagram showing the log deviation from initial level of the lymphocyte count, esterase-positive cells, polymorphonuclear leucocyte count, and PHA mitotic response.

our earlier observation on patients in this trial (Gill *et al.*, 1977*a*) and those reported by other workers (Minton *et al.*, 1976). The pattern of fluctuation of polymorphonuclear leucocytes and lymphocytes is reminiscent of that seen after i.v. injection of prednisolone (Clarke *et al.*, 1977); consisting of simultaneous polymorphonuclear leucocytosis and lymphopenia. This dual effect of CP is delayed by 20 h, and is possibly mediated through the endogenous release of corticosteroids from the adrenals.

The reduced mitogenic response to PHA reflects the loss of lymphocytes and possibly monocytes from the peripheral blood, since the latter have been shown to

FIG. 8.—Summary diagram showing the log deviation from initial level of the lymphocyte count and the cytotoxicity assays.

be involved in this response (Oppenheim *et al.*, 1968; Potter & Moore, 1977). However, the fall in antibody- and PHA-induced lymphocytotoxicity 3 h after CP infusion is considerably greater than could be expected from the drop in lymphocyte counts alone.

The i.v. administration of prednisolone produces a nonsynchronous pattern of loss of lymphocytes and their cytotoxicity similar to that found here. However, the maximal depression of antibody-induced cytotoxicity by steroids occurs 20 h after the lowest lymphocyte count. Although a further loss of cytotoxicity may have occurred 48 h after CP administration, the first significant fall, which was measured at 4 h, cannot be ascribed to the effect of endogenous steroids. A more likely explanation is that K cells are temporarily blocked by circulating products resulting from the infusion, such as antigen–antibody complexes formed between the bacteria and the natural antibodies which are present in these patients. All the patients studied were receiving their first infusion, and a low level of such antibodies might be expected. However, K cells have been shown to be very sensitive to the blocking action of such complexes (MacLennan, 1972). Fibrinogen degradation products can also interfere with the expression of lymphocyte responses *in vitro* (Ginmann *et al.*, 1976) and these products are markedly elevated 3 h after infusion of CP (Cederholm-Williams *et al.*, 1978) when the mitogenic and cytotoxicity responses were reduced. Further experiments using isolated lymphocytes are clearly required to determine whether alterations in lymphocyte subpopulations or serum factors are responsible for these changes.

Thatcher & Crowther (1978) have demonstrated an initial fall followed by a rise in cytotoxicity after CP, but differences in the time at which samples were assayed and in the methods of quantitating cytotoxicity make direct comparisons with their results difficult. Similar considerations apply to related studies in cancer patients (Webster *et al.*, 1978) in whom the mitogenic response to PHA was found to be unchanged.

The clinical relevance of the suppression of lymphocyte cytotoxic and mitogenic activities during the 24 h after CP treatment is uncertain, but it is of interest in this regard that there was a significant incidence of herpetic and varicelliform eruptions in these patients within 24–36 h of infusion (Gill *et al.*, 1977*b*). One potentially important practical point which stems from these findings concerns the scheduling of CP in clinical protocols. It would seem desirable to design the frequency of CP therapy in order to allow immunological recovery between treatments, though further studies of such protocols would be required to confirm this suggestion.

The effect of the monocyte series needs comment, since it has been suggested in several animal tumour systems that CP produces its anti-tumour effects by increasing both macrophage numbers (Baum & Fisher, 1972; Wolmark & Fisher, 1974) and phagocytic function (Scott, 1972*b*). The rapid reduction in monocyte number immediately after infusion is similar to the findings of other workers (Minton *et al.*, 1976) but we also observed a significant increase 7 days later.

There were also qualitative differences between the cells of the monocyte series observed at these times, those present 24 h after treatment being smaller and with fewer esterase-positive granules than those present at 7 days. This, together with the subsequent increase in numbers, is in accord with a stimulatory effect of CP on marrow which has been documented in mice (Chare & Baum, 1978; Foster, 1978) and rats (Wolmark & Fisher, 1974). The timing of these changes closely parallel those observed in mice, in which increased plasma levels of marrow colony-stimulating factor were detected within hours of CP injection (Foster, 1978; Eliopoulos *et al.*, 1978). Considered in conjunction with the acute suppression and recovery in lymphocyte function, the increased monocyte numbers after 7 days suggest that one rational schedule for CP therapy might be infusions at 1–2-weekly intervals, rather than the monthly schedule which is currently widely used clinically. This suggestion would require confirmation by further studies, but Hedley *et al.* (1979) have recently published data on monocyte function which also suggest that administration more often than every 4 weeks may be more appropriate in humans. These workers also demonstrated increased monocyte function using small intradermal doses of CP.

The increase in serum lysozyme levels was significant, but transient. This enzyme is present in both polymorphs and macrophages, but recent clinical and experimental studies have proposed that its measurement provides one parameter of macrophage-mediated defence against tumours (Currie, 1976). Its infusion does not follow the change in numbers of monocytes, and the changes observed probably represent the rapid release from mature cells of the granulocyte and macrophage series. The increase in lysozyme does, however, closely parallel the enormous increase in fibrinolytic activity which we have observed in these patients (Cederholm-Williams *et al.*, 1978) and it is interesting to note that both lysozyme and plasminogen activators are important secretory products of macrophages (Gordon, 1976).

Although we have demonstrated consistent transient suppression of lymphocyte function in the blood of these patients immediately after infusion, this should not necessarily be regarded as a contraindication to such immunotherapy. We take this view because of the rapid recovery which occurs; because no sustained immunosuppression was demonstrable in patients who had had multiple infusions (Waller *et al.*, 1980); and because in animal tumour systems CP still exerts its anti-tumour effect, despite concomitant immunosuppression.

We are grateful to Mr Brian Walker for skilled technical assistance and to Miss E. J. Howes for carrying out the white-cell counts and differentials. We are also indebted to Dr S. Gordon for helpful discussion.

REFERENCES

ALLWOOD, G. G. & ASHERSON, G. L. (1973) Depression of delayed hypersensitivity by pretreatment with Freund type adjuvants. III Depressed arrival of lymphoid cells at recently immunized lymph nodes in mice. *Clin. Exp. Immunol.*, **2**, 579.

BAUM, M. & FISHER, B. (1972) Macrophage production by the bone marrow of tumor-bearing mice. *Cancer Res.*, **32**, 2813.

CASTRO, J. E. (1977) Effects of *Corynebacterium parvum* on tumour metastases in mice. *Br. J. Surg.*, **64**, 721.

CEDERHOLM-WILLIAMS, S. A., KING, A., ALLINGTON, M. J., GILL, P. G., SHARP, A. A. & BRITTON, B. J. (1978) Coagulation and fibrinolysis during the infusion of *Corynebacterium parvum* in man. *Br. J. Cancer*, **37**, 1074.

CHARE, M. J. B. & BAUM, M. (1978) The effect of *Corynebacterium parvum* on the proliferation of monocyte precursors in the bone marrow of mice. *Dev. Biol. Stand.*, **38**, 195.

CLARKE, J. R., GAGNON, R. F., GOTCH, F. M. & 4 others (1977) The effect of prednisolone on leucocyte function in man: A double-blind controlled study. *Clin. Exp. Immunol.*, **28**, 292.

CURRIE, G. A. (1976) Serum lysozyme as a marker of host resistance: II. Patients with malignant melanoma, hypernephroma and breast carcinoma. *Br. J. Cancer*, **33**, 593.

ELIOPOULOS, G., ANDRE, S. & HALPERN, B. (1978) Monocytosis inducing activity (MIA) of serum in *Corynebacterium parvum* treated mice. *Dev. Biol. Stand.*, **38**, 183.

FOSTER, R. (1978) Effects of *Corynebacterium parvum* on granulocyte macrophage production and toxicity of chemotherapy. *Dev. Biol. Stand.*, **38**, 245.

GALE, D. G. L. & MACLENNAN, I. C. M. (1976) A method of measuring antibody and phytohaemagglutinin induced lymphocyte dependent cytotoxicity using whole blood. *Clin. Exp. Immunol.*, **23**, 252.

GILL, P. G., WALLER, C. A., CLARKE, J., DARLEY, J., & MORRIS, P. G. (1977*a*) The effect of *Corynbacterium parvum* on human effector cells in peripheral blood. *Dev. Biol. Stand.*, **38**, 455.

GILL, P. G., MORRIS, P. J. & KETTLEWELL, M. (1977) The complications of intravenous *Corynebacterium parvum* infusion. *Clin. Exp. Immunol.*, **30**, 229.

GINMANN, G., PEES, G., SCHWARVE, G. & SCHEURLEN, P. G. (1976) Immunosuppression by micromolecular fibrinogen degradation products in cancer. *Nature*, **259**, 399.

GORDON, S. (1976) Macrophage neutral proteinases and chronic inflammation. *Proc. Natl Acad. Sci.*, **278**, 176.

HEDLEY, D. W., NYHOLM, R. E. & CURRIE, G. A. (1979) Monocytes and macrophages in malignant melanoma: IV Effects of *C. parvum* or monocyte function. *Br. J. Cancer*, **39**, 558.

MACLENNAN, I. C. M. (1972) Competition for receptors for immunoglobulin on cytotoxic lymphocytes. *Clin. Exp. Immunol.*, **10**, 275.

MACLENNAN, I. C. M., CAMPBELL, A. C. & GALE, D. G. L. (1976) Quantitation of K-cells. In *In vitro Methods in Cell-mediated Immunity*. Ed. Bloom & David. Academic Press. p. 511.

MAINI, R. N., CLARKE, B., ROFFE, L. & LEONARD, R. B. (1973) A micro-technique for measurement of lymphocyte transformation using whole blood. *Proc. 8th Leucocyte Culture Conference*. Uppsala. p. 129.

MINTON, J. P., ROSSIO, J. L., DIXON, B. & DADD, M. C. (1976) The effect of *Corynebacterium parvum* on the humoral and cellular immune systems in patients with breast cancer. *Clin. Exp. Immunol.*, **24**, 441.

OPPENHEIM, J. J., LEVANTHAL, B. G. & HERSH, E. M. (1968) The transformation of column purified lymphocytes with non-specific and specific antigenic stimuli. *J. Immunol.*, **101**, 262.

OSSERMAN, F. F. & LAWLAR, P. D. (1966) Serum and urinary lysozyme (muraminidase) in monocytic and monomyelocytic leukemia. *J. Exp. Med.*, **124**, 921.

POTTER, M. R. & MOORE, M. (1977) The effect of adherent and phagocytic cells on human lymphocyte PHA responsiveness. *Clin. Exp. Immunol.*, **27**, 159.

REED, R., GUTTERMAN, J. A., MAVLIGIT, G. M., BURGESS, A. A. & HERSH, E. M. (1975) Preliminary report of a Phase I clinical and immunological study in cancer patients. In *Corynebacterium parvum*. p. 349.

SCOTT, M. T. (1972*a*) Biological effects of the adjuvant *Corynbacterium parvum*. I. Inhibition of PHA, mixed lymphocyte and GUH reactivity. *Cell Immunol.*, **5**, 459.

SCOTT, M. T. (1972*b*) Biological effects of the adjuvant *Corynbacterium parvum*. II. Evidence for macrophage—T cell interaction. *Cell Immunol.*, **5**, 469.

SCOTT, M. T. (1974) Corynbacterium as a therapeutic anti-cancer agent *Semin. Oncol.*, **1**, 367.

THATCHER, N. & CROWTHER, D. (1978) Effects of BCG and *Corynebacterium parvum* on immune reactivity in melanoma patients. *Dev. Biol. Stand.*, **38**, 447.

WALLER, C. A., CAMPBELL, A. C. & MACLENNAN, I. C. M. (1976) Two populations of lymphocytes involved in phytohaemagglutinin-induced cytotoxicity of a dividing target cell. *Scand. J. Immunol.*, **5**, 931.

WALLER, C. A. & MACLENNAN, I. C. M. (1977) Analysis of lymphocytes in blood and tissues. In *Techniques in Clinical Immunology*. Ed. Thompson. Oxford: Blackwell. p. 170.

WALLER, C. A., GILL, P. G. & MACLENNAN, I. C. M. (1980) Enhancement of lymphocyte mediate cytotoxicity after tumour resection in patients with colorectal cancer. *J. Natl Cancer Inst.* (In press.)

WEBSTER, D. J. T., CHARE, M. J. B. & BAUM, M. (1978) The effect of intravenous infusion of *Corynebacterium parvum* on an immune profile of women with breast cancer. *Dev. Biol. Stand.*, **38**, 467.

WOLMARK, N. & FISHER, B. (1974) The effect of a single and repeated administration of *Corynebacterium parvum* on bone marrow macrophage colony production in syngeneic tumour-bearing mice. *Cancer Res.*, **34**, 2869.

YAM, L. T., LI, C. Y. & CROSBY, W. H. (1971) Cytochemical identification of monocytes and granulocytes. *Am. J. Clin. Pathol.*, **55**, 283.